# Receptor for Advanced Glycation End Products Acts as a Fuel to Colorectal Cancer Development

**DOI:** 10.3389/fonc.2020.552283

**Published:** 2020-09-29

**Authors:** Fatemeh Azizian-Farsani, Navid Abedpoor, Mohammad Hasan Sheikhha, Ali Osmay Gure, Mohammad Hossein Nasr-Esfahani, Kamran Ghaedi

**Affiliations:** ^1^Department of Medical Genetics, Shahid Sadoughi University of Medical Sciences, Yazd, Iran; ^2^Department of Animal Biotechnology, Cell Science Research Center, Royan Institute for Biotechnology, Academic Center for Education, Culture and Reasearch (ACECR), Isfahan, Iran; ^3^Department of Molecular Biology and Genetics, Faculty of Science, Bilkent University, Ankara, Turkey; ^4^Division of Cellular and Molecular Biology, Department of Cell and Molecular Biology and Microbiology, Faculty of Biological Science and Technology, University of Isfahan, Isfahan, Iran

**Keywords:** AGEs, advanced glycation end products, CRC, colorectal cancer, damage-associated molecular pattern molecules (DAMPs), pattern recognition receptor (PRR), RAGE (receptor for advanced glycation end products), tumourogenesis

## Abstract

Receptor for advanced glycation end-products (RAGE) is a multiligand binding and single-pass transmembrane protein taken in diverse chronic inflammatory conditions. RAGE behaves as a pattern recognition receptor, which binds and is engaged in the cellular response to a variety of damage-associated molecular pattern molecules, as well as HMGB1, S100 proteins, and AGEs (advanced glycation end-products). The RAGE activation turns out to a formation of numerous intracellular signaling mechanisms, resulting in the progression and prolongation of colorectal carcinoma (CRC). The RAGE expression correlates well with the survival of colon cancer cells. RAGE is involved in the tumorigenesis, which increases and develops well in the stressed tumor microenvironment. In this review, we summarized downstream signaling cascade activated by the multiligand activation of RAGE, as well as RAGE ligands and their sources, clinical studies, and tumor markers related to RAGE particularly in the inflammatory tumor microenvironment in CRC. Furthermore, the role of RAGE signaling pathway in CRC patients with diabetic mellitus is investigated. RAGE has been reported to drive assorted signaling pathways, including activator protein 1, nuclear factor-κB, signal transducer and activator of transcription 3, SMAD family member 4 (Smad4), mitogen-activated protein kinases, mammalian target of rapamycin, phosphoinositide 3-kinases, reticular activating system, Wnt/β-catenin pathway, and Glycogen synthase kinase 3β, and even microRNAs.

## Introduction

Around 147,950 persons will be identified with colorectal carcinoma (CRC), and 53,200 will die of it by the year 2020, with 17,930 cases and 3,640 deaths in people younger than 50 years. CRC has a complex and multifactorial etiology, strictly related to environmental and genetic factors, including adenomatous polyposis coli (APC) alteration, long-lasting inflammation, metabolic diseases, and also gut microbiota alteration (1–5).

Genetic and epigenetic abnormalities within multistep procedures known as carcinogenesis progressively transform healthy human cells into highly malignant derivatives. Six alterations in the cell physiology essential for malignant progression include the ability to invade and metastasize, bypassing of apoptosis, independence in growth factors, constant angiogenesis, unlimited replicative potential, and insensitivity to growth-inhibitory signals. Moreover, inflammation context, genomic instability, immune devastation, and reprogramming of energy metabolism are hallmarks of cancer (6, 7). The molecular signaling of CRC development, limited diagnosis, and therapy of this lethal disease remain mainly unclear. In this regard, chronic inflammation is broadly considered as an essential factor underlying CRC development.

**Figure 1:**
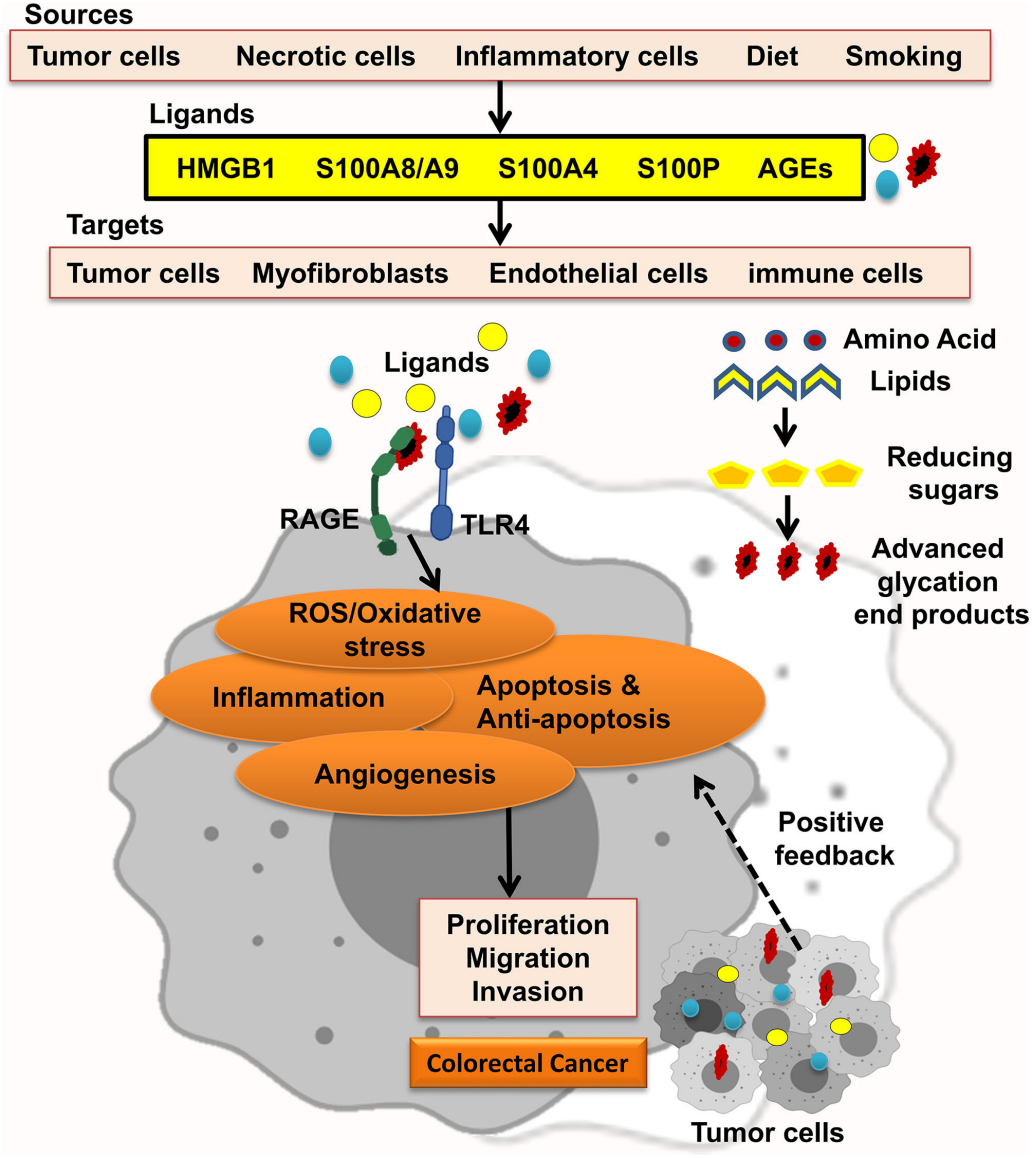
RAGE signaling promotes CRC. Different RAGE ligands are released from several types of cells and start RAGE activation, which enhances ROS/oxidative stress, inflammation, apoptosis and antiapoptosis, and angiogenesis and led to proliferation, migration, and invasion of CRC tumor cells.

The receptor for advanced glycation end-products (RAGE) pathway is seriously involved in the pathologies of different cancers. RAGE was first explored as a cell surface receptor for advanced glycation end-products (AGEs). AGEs was also the first ligand recognized for RAGE. Accumulation of AGEs and the other ligands is related to chronic inflammation; hence, RAGE signaling is regarded as a fundamental pathway in inflammation-related disorders. In this regard, inflammation has been well-recognized to take in CRC initiation and progression. In agreement, many studies showed that RAGE signaling has been involved in colitis-associated colon carcinogenesis. Notably, Heijmans et al. recognized that loss of RAGE in mice model prohibited sporadic progression of intestinal adenomas. Studies using CRC cell lines also discovered that RAGE signaling is strongly related to abundant malignant behavior of CRC cells, including chemoresistance, invasion, and proliferation. These findings proposed that RAGE plays a key role in the connection between inflammation and colon carcinogenesis (Figure 1) (8–11).

RAGE ligands, for example, AGEs, exist in modern diet mostly in fast foods and processed foods. Briefly, AGEs form in foods high in protein and fat, which brown through cooking under high temperatures. Also, exposure to dry high temperature produces more AGEs than cooking in liquid. Thus, grilling, broiling, and frying meats generate more AGEs than boiling. For instance, 15 min of broiling a chicken breast form AGEs five times higher than the same food boiled for 1 h. Cigarette smoking is another source of AGEs. It is known that tobacco curing results in forming AGEs (12). Considerably, a diet high in sugar and fat and highly processed, besides an inactive lifestyle, also results in AGE accumulation which contributes to chronic disease development and complications. It has been shown that cancer-associated cell signaling cascades such as MAPK (mitogen-activated protein kinase) and AKT (protein kinase B) are activated through rises of AGE accumulation pool, leading to aberrant cellular functions. As in breast cancer, a link between esterogen receptor (ER) status and AGE levels was observed in tumor and serum samples (13). Thus, changing the lifestyle would decrease AGE burden to promote healthy aging.

This review was assumed to elucidate recent knowledge of RAGE–ligand interaction, the ligand sources, and related tumor markers, taking out from clinical studies and downstream cascades in the context of inflammation, type 2 diabetes (T2D), and also therapeutic strategies, particularly in CRC, and identification of RAGE signaling pathway. The significant role of RAGE signaling pathway in cancer suggests a brilliant molecular target for CRC treatment, especially antimetastatic therapeutics.

## RAGE

RAGE is a kind of the immunoglobulin protein family with an immunoglobulin-like receptor structure resembling cell surface molecules (14, 15). Furthermore, different inflammation-related molecules, including HMGB1, S100A/calgranulins, α-amyloid, and AGEs, could activate RAGE (16). The localization of RAGE gene is on chromosome 6, and it is close to the dominant histocompatibility complex class III region in humans and mice. The long RAGE mature is 382-amino-acid residues, composed of five domains including extracellular domains such as V-domain, which attaches to ligands, C1 and C2 domains, a single transmembrane spanning helix, and a short cytosolic region, which is an important domain for successful RAGE signaling. RAGE contains two N-glycosylation sites. One is located inside the V-domain, and another adjacent to the V-domain forming oligomers at the cell surface. RAGE contains two N-glycosylation sites. One is located inside the V-domain, and another adjacent to the V-domain (17–19).

Based on numerous pathological conditions, splicing variants of RAGE have been categorized and named. Moreover, the RAGE gene includes 11 introns/exons that could be spliced into the diverse variants at the DNA level. Indeed, RAGE is classified into three primary isoforms, including Nt-RAGE (N-terminally truncated isoform RAGE_v2), the full-length RAGE, and secreted RAGE, which acts as a “decoy receptor” that might be inhibiting RAGE ligands from interacting with cell surface RAGE and is named sRAGE (secretory C-truncated RAGE) (20).

### Role of RAGE in Normal Physiology and Different Pathologies

RAGE is expressed in diverse cells, mature or immature, including macrophages, monocytes, endothelial cells, fibroblasts, and smooth muscle cells. As throughout the development stages the RAGE expression level is enhanced, principally in the brain, nevertheless, the expression level of RAGE declined in adult tissues, as the activation of RAGE in fibroblasts related to the proliferation and migration of fibroblasts in the tumor microenvironment (21). Moreover, compared to other tissues, high RAGE expression is seen in lungs during embryonic development (22–24). So, it has been proposed that RAGE may show properties limited only to the pulmonary environment owning a number of significant physiological roles comprising modulation of cell spreading, adhesion to ECM parts, proliferation, and migration (25).

Also, the upregulation of RAGE is seen during diverse pathological events, including inflammatory bowel disease (IBD), stroke, sepsis, diabetes, renal disease, Alzheimer disease (AD), heart failure, osteoarthritis, atherosclerosis, peripheral vascular disease, psoriasis, rheumatoid arthritis, Takayasu arteritis, and cancer (17, 21, 26–28).

The interaction of RAGE with its diverse ligands can mediate multiple physiological and pathological functions, including inflammation, oxidative stress, neurodegeneration, maintenance of homeostasis, tumorigenesis, promotion of neurite outgrowth, cell survival, cell migration, and neuronal differentiation (29). Furthermore, in skeletal muscle tissue, RAGE expression is developmentally regulated. RAGE can be detected in immature, nearly mature, and some mature myofibers up to 11 days after birth in rodents, with RAGE expression being restricted to the sarcolemma (30). In this regard, the effect of prolonged RAGE blockade in human subjects is important because RAGE plays vital roles in normal physiology (31). The ligands for RAGE in the central nervous system (CNS) include AGEs, b-amyloid peptides, amphoterin (HMGB1), and S100B protein. As in AD, AGEs and b-amyloid activate RAGE. Several studies, reviewed by Ramasamy et al. (32), have shown that RAGE-dependent modulation of gene expression and cellular properties is dependent on the binding of different ligands for RAGE, each of which activates distinct downstream signaling pathways and elicits cell type–specific effects (26, 27, 32). So as regards tissue-specific RAGE ligand expression, the roles of RAGE vary in different pathologies, and also therapeutic strategies change, as in breast cancer, S100A7 is a promising target therapy but not in colorectal cancer. Moreover, RAGE expression in all the cancers is increased, but in lung cancer, the expression is decreased (26).

### RAGE in Cancer

Many studies have been shown that RAGE is a member of a gene family related to the invasion-genes. There are a close association between RAGE upregulation and etiology of different cancers, including breast, prostate, lung, esophageal, and colorectal cancer. RAGE–ligand interaction leads to increased proliferation, migration, and metastatic nature of tumor cells (33–39). Moreover, Dahlmann et al. in 2014 and Shimomoto et al. in 2012 have found that in the colon and oral carcinogenesis, the expression of RAGE is increased (40–43). While most of normal tissues express low level of RAGE, it might be enhanced in inflammatory conditions as a cellular response. In line with this, inflammation in the intestinal tract, due to T2D or obesity, leads to amplified adenoma formation. These results were consistent with the findings of Luo et al. which indicated progression and tumor formation, significantly increased by upregulation of RAGE expression in CRC. Moreover, RAGE expression increased in IBD, which is a CRC risk factor (9, 44–47). Liang and colleagues in 2011 have assumed that the amplified density of microvessel was associated with RAGE expression in colorectal cancer, and they claimed that knockdown of RAGE could be prevented by invasion capacity, whereas cell viability did not significantly affect SW480 cells (48). In contrast, some studies have shown that in normal lung tissue and non-small cell lung carcinoma the expressions of HMGB1 and RAGE are increased. Therefore, there is a negative association between RAGE and disease prognosis. However, RAGE declines the malignant phenotypes of the disease, which is associated with differentiation rhabdomyosarcoma and myogenic of myoblasts. In addition, RAGE expression is increased in esophageal cancer. These findings show that the role of RAGE depends on the tissues that are involved and the types of cells (49–53).

Ligand–RAGE is related in a paracrine and autocrine manner in the tumor microenvironment to stimulate cell migration, survival, and invasion (54). Clinical studies have indicated that RAGE is associated with its protumoral property. Moreover, RAGE is relocated to the cytoplasmic membrane during progression from colonic adenoma to colonic adenocarcinoma. In contrast, Kostova et al. in 2010 have found that high-grade cancer could show non-specific RAGE localization (42, 55).

RAGE–ligand (ligand secreted from tumor cells or by nearby tissues) interaction happens in the extracellular tumor microenvironment. While the RAGE expression is high in a tumor, high endogenous ligand expression is not essential for activation of the receptor (56).

Furthermore, Gly82Ser, which is the single-nucleotide polymorphism (SNP) of RAGE (rs2070600), enhances ligand binding to boost the downstream signaling pathway, which is associated with amplified risk of numerous cancer types; particularly, the frequency of this polymorphism is much higher in late-stage CRC patients (57, 58). Thus, polymorphism of RAGE can affect its function.

## Ligands

### Advanced Glycation End-Products

AGEs are crosslinking compounds formed through non-enzymatic glycosylation between reducing sugars and free amino groups of lipids, proteins, and nucleic acids. Likewise, AGEs have two kinds of forms: endogenous and exogenous (59). To date, well-identified AGEs comprise six subtypes, such as glucose-derived AGEs (AGE-1), glyceraldehyde-derived AGEs (AGE-2), glycolaldehyde-derived AGEs (AGE-3), methylglyoxal (MG)–derived AGEs (AGE-4), glyoxal-derived AGEs (AGE-5), and 3-deoxyglucosone-derived AGEs (AGE-6) (60). Foods are the other source of AGEs (e.g., fast foods, meat, bread, daily products), and their formation is induced by food processing at high temperature, besides different ways such as food store for a long time or additive usage, which amplifies AGEs in the foods (61). Interestingly, the other rich source of AGEs is cigarettes. Jiao and colleagues in 2011 have shown that the plasma level of sRAGE in Finnish male smokers significantly diminished (62).

Many studies have demonstrated AGEs are considered as potent toxic molecules that produce several non-communicable diseases such as T2D, CRC, cardiovascular diseases, Parkinson disease, AD (63–68). Epidemiological evidence has reported that elevated AGEs were associated with CRC (69).

Interestingly, Shimomoto et al. reported that in Western diet–fed rats [15% linoleic acid (LA) diet with 10% glucose drink], significantly aberrant crypt foci (ACFs) were boosted. In line with this, in another study, it has been shown that AGEs might develop CRC in rats that consumed Western diet (41).

The primary mechanism by which AGEs provoke biological function is through binding to RAGE, which triggers activation of adverse cellular effects contributing to enhancing oxidative stress, inflammation, and tumorigenesis. AGEs or different kinds of ligands bind to sRAGE as acting “receptor decoy” that could inhibit RAGE signaling pathways. Studies have shown that AGEs accumulate in the extracellular of different tissue due to promotion of chronic disease (70, 71).

Moreover, intensive studies have indicated that subtypes of AGEs induced the metastasis and invasion in patients with CRC. Deng and colleagues in 2017 have found that glucose-derived AGEs, which is a critical subtype of forms of AGEs, induced the metastasis and invasion in patients with CRC (72). Moreover, another subtype of AGEs is glyceraldehyde-derived AGEs that are mediated proinflammatory and pro-oxidative in CRC. Nevertheless, there is controversy in the results; for example, Kong et al. in 2015 have evaluated that glyceraldehyde-derived AGEs were increased in CRC patients, but not in association with colon cancer. Indeed, Kong et al. claimed there is close association with the risk of rectal cancer (69). In agreement with these data, Liang et al. demonstrated RAGE could be associated with increasing microvessel density, invasion capacity, cell viability, and angiogenesis, and knockdown of RAGE repressed expression of vascular endothelial growth factor (VEGF) and SP1 protein in colorectal cancer cells (48).

Likewise, Wang et al. have indicated AGEs might upregulate oncogenes (73).

Another component involved in AGE formation is MG. This compound is produced during glycolysis as a side product. A study by Chiavarina et al. predicted that MG accumulated through higher glucose metabolic rates in tumors (74). Furthermore, Lin et al. in 2018 found that MG had a crucial role in colon cancer progression. Additionally, MG could mediate low-grade carbonyl stress and lead to oxidative stress and inflammation and promote tumor growth, and the degree of malignancy of tumor cells is enhanced (75). On the other hand, exogenous AGEs might increase the amount of glycotoxins, which impair many metabolic processes, especially, cancer development. Sakellariou et al. have assumed AGEs/RAGE increased detoxification enzyme glyoxalase (GLO), and there was a relationship between exogenous AGEs and glycotoxins in CRC patients (76).

### HMGB1

*HMGB1* gene encodes HMGB1/amphoterin, a non-histone chromosomal structural protein (77). HMGB1 is isolated as a 30-kDa cytosolic heparin-binding protein in growing brain tissue and is related to outgrowth neurite. HMGB1 has diverse functions in the cytoplasm, extracellular milieu, and nucleus. Moreover, HMGB1 binds to a type of non-B DNA type in the nucleus and contributing to several procedures, including recombination, replication, transcription, stability of genomic, and DNA repair (78). Furthermore, in the cytoplasm, HMGB1 is related to motility of cell as noticed in outgrowing neurites. Moreover, HMGB1 in motile cell accelerates the formation of adhesion molecules, actin–polymer formation, and filopodia, in addition to detachment from the extracellular matrix. Fages et al. have shown that the mechanism of HMGB1 is similar to that of outgrowing neurites on cell migration in cancer cells (79). HMGB1 expression is high in immature cells and malignant cells and has the main role of regulating of cell migration function (80).

HMGB1 has different molecular roles in cancer. HMGB1 promotes the expression of cellular inhibitor of apoptosis-2, a target gene of activated nuclear factor-κB (NF-κB), and restricted activation of apoptosomal caspase-9. As result, based on these data, HMGB1 might play an antiapoptotic role in colon cancer and decrease anticancer immune responses by stimulated apoptosis in immune cells (81). Notably, Tang et al. in 2010 have indicated endogenous HMGB1 activates an autophagy signal, which promotes cell survival (82). Interestingly, HMGB1 also has a cytokine function that has an extranuclear role when it is inactively released from necrotic and tumor cells after radiotherapy and chemotherapy or actively from monocytes and macrophages into the extracellular environment (83). HMGB1 expression and secretion are unregulated in response to the stimulation of cells by endotoxin, proinflammatory cytokines, platelet activators, and oxidative stresses in macrophages. These results have supported a paracrine/autocrine mechanism for the amphoterin/RAGE action detected in CRC cells (80, 84). Moreover, DiNorcia et al. in 2010 and Heijmans et al. in 2012 have demonstrated the prompt of Lin cytokines; cellular stresses and growth factors involving deoxycholic acid and AGEs could amplify expression of HMGB1 in colon adenomas and carcinomas. In addition, studies have shown that upregulation of HMGB1 and RAGE has been linked with poor prognosis, metastasis, and tumor invasion in colorectal cancer. Based on intensive evidence, the main receptors of HMGB1 could be RAGE and toll-like receptors (TLR)-2 and TLR-4. In line with this, Harada and colleagues in 2007 have found that a specific receptor of HMGB1 was RAGE, and complex of HMGB1/RAGE could mediate abundant biological responses, including angiogenesis, axonal sprouting promotion, and outgrowing neurite and immune cell recruitment to an inflammatory place. Thus, it would be interesting to know which pathways of RAGE are activated by HMGB1 in colorectal cancer (45, 85–88).

Furthermore, in multiple ways, HMGB1 could be modified posttranslationally, which might determine the secretion and location of HMGB1 and bind to proteins and DNA. The difference in bioactivities of HMGB1 might be related to tissue sources or different cell types or its responses to different stimuli (89, 90).

### S100 Family

S100 is a member of proteins with low molecular weight (9–13 kDa), which is expressed in vertebrates, including at least 25 relatively non-ubiquitous calcium-binding proteins. Their functions depend on calcium concentration and could be changed. Besides, several studies focused on S100 proteins functions including, at the intracellular level, regulation of cell cycle, motility, differentiation, proliferation, apoptosis, Ca^2+^ homeostasis, cellular signaling, and energy metabolism. In addition, S100 has another role that regulated a variety of intracellular actions, such as cytoskeletal function, protein phosphorylation, and defense from oxidative cell injury. Interestingly, S100 proteins could be active via surface receptors in paracrine and autocrine manner at the extracellular level. As a result, S100 could be able to activate signaling pathways at these sites of chronic inflammation via peripheral blood mononuclear cells and macrophages, including T lymphocytes and RAGE endothelial cells. Diverse S100 proteins have been documented and have expression in different tissues such as various peripheral tissues in a cell-specific manner. S100 proteins remained in their free form at low calcium state and bind calcium and undergo a specific conformational change after the inflow of calcium through voltage-gated or receptor-induced channels, which results to an adjustment of their hydrophobic surface properties. Furthermore, calcium level elevation led to structure change of S100 proteins; consequently, changing structure could allow interacting with the hydrophobic regions of the target proteins (91–97).

Severe vibrant evidence has shown S100 is involved in a variety of biological events associated with carcinogenesis, such as a gene being located on human chromosome 1q21, which is an area susceptible to genomic rearrangements; altered expression in many malignant disorders; and interaction with numerous proteins, which in carcinogenesis play a vital role. Also, S100 protein might affect target proteins such as p53, NF-κB, and β-catenin (98).

S100 family is regulated by epigenetic mechanisms. Lindsey et al. showed that curing the various medulloblastoma cell lines with DNA demethylation releases S100A3, S100A10, S10011, and S100P proteins (99). Also, micro-RNAs (mi-RNAs) regulate Sl00 protein expression; however, there are limited studies in this area, and more investigation is required for this to be elucidated. MiR-568 regulates the expression of NFAT5, a transcription factor that triggers S100A4 expression (100, 101). Moreover, it has been reported that S100A6 overexpression is induced after DNA hypomethylation in gastric cancer. Binding of acetylated histone H3 to a promoter in gastric cancer tissue has been seen when there are minor intensities of CpG methylation in the second exon and first intron of the S100A6 gene (102). Also, lower methylation of S100P gene promoter was seen in different prostate cancer cell lines (103).

### S100A8/A9

Both S100A8 and S100A9 are recognized as calgranulins MRP8 and MRP14 or A and B. S100A8/A9 functions as a heterodimer and has a concurrent expression, whereas the proteins are produced from distinct genes (104, 105). S100A8/A9 is expressed in various cells of myeloid lineage, containing monocytes, granulocytes, early-stage macrophages, neutrophils, myeloid-derived suppressor cells (MDSCs), cancer cells, and myeloid-derived suppressor cells. Ang et al. in 2010 have shown that the response S100A9 was better than S100A8 in cancer cells (104, 106–110). Calprotectin could play a significant role in immune responses and inflammatory signaling pathways such as endothelial adhesion of monocytes and neutrophil and chemotaxis for neutrophils and also can serve as a marker of inflammation (93, 111). Additionally, neutrophils are released chemoattractants as inflammation, which is induced by extra involvement of inflammatory cells to damage the tissue (112). S100A8 and S100A9 are expressed in initially recruited monocytes; as they mature, they sustain S100A9 and lose S100A8 expression (113). In one study, the expression of HMGB1 and S100A8 significantly was increased but not S100A9 in polyps compared with normal tissue (45). Calprotectin has been involved in a wide range of chronic inflammation complications such as rheumatoid arthritis, cystic fibrosis, transplant rejection, and tuberculosis (114) and likewise multiple sclerosis, IBD, and psoriasis (112, 115, 116). Indeed, calprotectin is common to consider as a biomarker. Moreover, calprotectin fecal levels are commonly measured to identify IBD.

Some investigations indicate S100A8/A9 upregulation in cancer. This inflammatory context promotes tumorigenesis, metastasis by mediating the tumor cells, and migration of monocytes to metastatic sites. Besides, expression of S100A8/A9 in endothelial cells and myeloid in premetastatic organs in response to transforming growth factor β, tumor necrosis factor α (TNFα), and VEGF expressed by distal primary tumors promotes homing of tumor cells to premetastatic niches (11, 117, 118). Growing studies have verified that heterocomplex of S100A8/S100A9 led to MDSC migration and mediated tumor cell invasion (119, 120). Elevated MDSCs and S100A9 were indicated in peripheral blood and tumor tissue from CRC patients. Circulation of MDSCs and S100A9 was positively related to each other and associated with neoplastic progression. Moreover, S100A9 induces and activates chemotaxis; however, it did not affect MDSC viability. On the other hand, S100A9 is involved in immunosuppression during CRC progression by regulating MDSCs. S100A8/A9–RAGE interaction has been revealed to stimulate MDSC migration and tumor growth (104, 119, 121, 122). Ghavami et al. in 2008 claimed that the influence of calprotectin was dependent on the concentration in tumor cells. Ghavami et al. have indicated increased S100A8/9 concentrations enhance the apoptosis in colon carcinoma cell lines. However, tumor cell growth and migration are stimulated in low concentrations (104, 109). Turovskaya et al. showed that risk of colorectal cancer in patients with IBD is amplified. Myeloid progenitors infiltrate into dysplasia sites in human adenoma and colon tumor tissue and secrete S100A8 and S100A9. The primary receptor of S100A8/A9 on the tumor cells is RAGE. Calprotectin might be bound to RAGE and activate NF-κB signaling pathway, which plays a pivotal role in colitis-associated carcinoma (11).

### S100A4

S100A4 (metastatin-1, calvasculin) is a multifunctional protein with localization in the extracellular space, cytoplasm, and nucleus. The S100A4 expression was highly in diverse cancer tissues, related to metastatic tumor development, particularly in CRC patients. Intensive evidence shows that the expression of S100A4 could be considered as a prognostic marker in CRC. Different studies have shown that expression of S100A4 in CRC-xenografted mice is associated with metastases progression. Likewise, increasing expression of S100A4 is often related to pathological disorders including metastasis formation, epithelial–mesenchymal transition (EMT), and tumor outgrowth (47, 123–129).

S100A4 drives metastasis formation in different ways. Interestingly, the motility of cancer cells could be mediated by intracellular interactions with tumor-suppressing proteins, as well as components of the cytoskeleton. Besides, the intracellular S100A4 might increase cell motility through interactions with the proteins of cytoskeleton, including non-muscle tropomyosin, non-muscle myosin II, and actin filaments. Additionally, liprin β1 interacts with metastatin-1 and promotes invasiveness of primary tumors, which leads to cell adhesion causing a migratory phenotype. Besides, S100A4 bound to p53, which increased the progression of a more aggressive cell phenotype and ended in a modulation of p53 transcriptional function. Moreover, S100A4 recruits the factors of the immune system, or remodeling the extracellular matrix in the tumor–stroma microenvironment aggravates metastasis formation. S100A4 is released into extracellular space and stimulates endothelial cell motility and interacts with annexin II on the surface of endothelial cells, which enables angiogenesis and activates matrix metalloproteinase expression, which cleaves proteins of the extracellular matrix and thereby facilitates cell invasion into the adjacent tissues (93, 130–136).

### S100P

S100P was first purified in 1992 as a 95-amino-acid protein from human placenta, with a partial cellular spreading (137). It has been confirmed that elevated expression of S100P and then interaction with RAGE induce metastases, cell proliferation, and tumor invasion, like in colon cancer. Indeed, evidence has indicated S100P is among three signature genes that induce liver metastasis in a mouse model of colorectal cancer. In contrast, suppression of S100P inhibited colon cancer metastasis and growth, although ameliorated mice survival (8, 138, 139). Research has shown that S100P is augmented in inflammatory disorders such as IBD (8). Prostaglandin E_2_, which is augmented in epithelial CRC cells, could unregulate S100P. Notably, the S100P protein expression was related to the localization of the primary CRC tumor: the levels of S100P expression were elevated from the right to the left, being the maximum in the rectal carcinomas. Kaplan–Meier has investigation shown that elevated expression of S100P led to decrease of survival time of patients with CRC stages I, II, and III. These results have revealed there was a correlation between expression protein of S100P and survival time (140, 141).

## Clinical Studies

Table 1 summarizes clinical studies in CRC patients. It is well-recognized that CRC screening and monitoring strategies such as diagnosis and elimination of premalignant lesions and adenomatous polyps result in reduction of CRC mortality (155). Several clinical studies have pursued to carry out biomarkers that could be identified as potent metastasis diagnosis in patients with colorectal cancer. Kuniyasu et al. in 2003 have investigated 119 non-diabetic patients with CRC, who found out RAGE expression was increased in 55% of the cases. Besides, Sasahira et al. have investigated 96 colorectal adenomas and demonstrated that RAGE expression was significantly increased in adenomas with severe atypia, especially with membranous pattern (55, 156). Likewise, Liu and colleagues in 2014 studied the cases of 21 colorectal cancer patients with metabolic syndrome. They assessed circulating interleukin-6 (IL-6), VEGF, fasting insulin, and tumor expression of insulin-like growth factor-1 receptor (IGF-1R), insulin receptor (IR), insulin-like growth factor-1 (IGF-1), and RAGE markers before surgery and 6 months after tumor surgery. Liu et al. have indicated no differences in some circulating cytokines such as IL-6, IGF-1, and VEGF-1 in patients with CRC. However, Liu et al. observed RAGE and IGF-1 expressions were as biomarker in tumor tissue. Furthermore, hyperinsulinemia might be associated with metabolic syndrome and colon cancer (149). Additionally, Tóth et al. in 2007 considered four SNPs such as TNFα−308 G > A, RAGE−429 T > C, HSP70-2−1267 A > G, and LTA 252 A > G in genomic DNA in 183 Hungarian patients with CRC, and they observed that RAGE−429C, TNFα−308A, HSP70-2−1267G, and LTA 252G (8.1AH) haplotype were more frequent (19.1%) in patients with CRC (153).

**Table 1:** Tumor marker expression in tumor tissue and plasma of CRC patients related to RAGE signaling pathway.

**References**	**CRC patients/samples**	**Biomarker**
Caruso et al. (142)	31	FN3K
Chen et al. (2016)	1,249	sRAGE
Chiavarina et al. (74)	102 (Tumors)	MG, GLO-I
Choi et al. (143)	46 (Plasma)	sRAGE and S100A12
Comstock et al. (144)	126 (Serum)	VEGF,C-peptide, sRAGE
Deng et al. (72)	160 (Tumors and serum)	Glucose-derived AGEs, RAGE, SP1 and MMP2
Fahmueller et al. (145)	49 (Serum)	HMGB1
Hamaguchi et al. (146)	62	TIMP-1, IL-8, and EN-RAGE (more overall survival)
Harada et al. (86)	50 (Tumor tissues)	Trophinin
Huang et al. (88)	30 (Tissue and serum)	TCTP, HMGB1
Huang et al. (88)	106	phospho-Drp1^Ser616^,RAGE-G82S polymorphism (rs2070600)
Huang et al. (121)	52 (China; tumor tissue and blood)	S100A9 and MDSCs
Jiao et al. (62)	29,133 (Finnish male smokers; serum)	sRAGE
Jiao et al. (147)	158 (plasma)	sTNF-αRI„ sTNF-αRII, sIL-6R, EGF, IFNα2, G-CSF, MCP1, TNFβ, VEGF, and decreased sRAGE
Kong et al. (69)	396 (Rectal cancer, serum)	Glycer-AGE
Kucukhuseyin et al. (148)	80 (Istanbul)	AGEs
Kuniyasu et al. (2003)	119 (Non-diabetic)	Coexpression of RAGE and amphoterin
Liu et al. (149)	21 (10 MS, 11 non-MS)	IGF-1R, RAGE, IR
Qian et al. (58)	90	RAGE gene Gly82Ser polymorphism
Royse et al. (150)	65 (Males; tumor epithelia	AGER, IL1A, IL6, MyD88, and TLR5
Sakellariou et al. (76)	133 (Colonic mucosa)	AGE, RAGE and GLO-I, AdipoR2
Sasahira et al. (55)	96	Membranous RAGE expression
Shen et al. (151)	90 (Tissue)	SOX9 and S100P
Shen et al. (152)	25	S100P
Tóth et al. (153)	183 (Hungarian)	TNF-a 2308A, RAGE 2429C, HSP70-2 21267G, LTA 252G (8.1AH) haplotype
Turovskaya et al. (11)	9 (Tumor tissues)	Carboxylated glycans, RAGE, and S100A8/A9
Zinkzuk et al. (154)	50	Catalase and malondialdehyde

Another biomarker that clinical studies focused on was sRAGE. According to evidence, some studies have indicated sRAGE was diminished in CRC. Chen et al. in 2016 have demonstrated that sRAGE was inversely correlated with body mass index (BMI). Also, among women with BMI ≥25 kg/m^2^, risk of CRC with the highest sRAGE concentration was significantly lesser compared to women with lower sRAGE concentration. On the other hand, the RAGE pathway might be a pivotal key in CRC with obesity in postmenopausal women (157). In another study, Choi and colleagues have examined the association between biomarkers and respiratory complications in 46 patients with CRC. This study has predicted that plasma sRAGE and S100A12 could be an appropriate biomarker for diagnosing the development of postoperative respiratory complications (143). Notably, Jiao et al. have studied the cases of 29,133 Finnish male smokers and found that increasing levels of serum sRAGE were associated with decreasing risk of CRC in male smokers (62). Also, Jiao et al. in 2012 observed plasma levels of sTNF-αRII, sTNF-αRI, TNFβ, IFNα2, sIL-6R, MCP1, epidermal growth factor (EGF), granulocyte colony-stimulating factor (G-CSF), and VEGF significantly increased; in addition, sRAGE level was decreased in 158 cases with colorectal adenoma (147). Furthermore, Comstock et al. in 2014 have found that serum concentrations of VEGF and C-peptide were amplified in 126 asymptomatic men (48–65 years old), and sRAGE was decreased but did not associate IGF-1 with polyp number or type. They assumed these biomarkers could benefit the diagnosis more than colonoscopy (144).

In contrast, Kong et al. assumed that elevated levels of glycer-AGEs were not significantly associated with colon cancer risk but had a positive association with the risk of rectal cancer (69). Interestingly, Hamaguchi and colleagues in 2019 have explored eight biomarkers that were associated with potential prognosis in 62 metastatic CRC patients who received aflibercept plus FOLFIRI. Consequently, they claimed tissue tenascin-C, IL-8, extracellular new receptor for advanced glycation end-products (EN-RAGE), pulmonary surfactant–associated protein D, tissue inhibitor of metalloproteinases 1 (TIMP-1), kallikrein 5, tumor necrosis factor receptor 2 (TNFR2), and IGF-binding protein 1 were identified as biomarkers potentially prognostic for overall survival. In addition, among these biomarkers, the lower the levels of TIMP-1, IL-8, and EN-RAGE (*P* < 0.001), the more overall survival was indicated (146). Fahmueller et al. in 2013 investigated a homogenous cohort of CRC patients with hepatic metastases and showed that after 24 h radioembolization (RE) therapy the serum levels of HMGB1 were enhanced, whereas RAGE and DNAse levels remained unchanged. So, HMGB1 is a valuable serum biomarker for early estimation of therapy response and prognosis in CRC patients with liver metastases undertaking RE therapy (145). Moreover, glucose promotes Millard reaction and develops adverse effects on exposed cells. In this regard, fructosamine 3 kinase (FN3K) enzyme was recognized as a repair protein in human tissues. Interestingly, Caruso et al. have found that 31 CRC patients had deficient FN3K gene expression, which resulted in significant detrimental effects of “sugar stress” on cell function in 31 CRC patients (142). Royse et al. indicated lower expression of AGER, IL1A, MYD88, and TLR5; however CXCL8 and S100P were higher in tumor epithelia, which was correlated with less survival (150).

Intensive researches have been made over the past decades to give insight into the relationship between reactive oxygen species (ROS) and CRC (158–160). Consistently, Zinkzuk et al. in 2019 carried out a study and showed superoxide dismutase was amplified, whereas the activities of catalase, glutathione peroxidase, and glutathione reductase were lower in 50 CRC patients. Zinkzuk et al. have assumed redox could be potential biomarkers for CRC diagnosis (154). Moreover, Sakellariou et al. confirmed that detoxification enzyme GLO-I expression was directly related to RAGE, but inversely related to AGEs in 133 primary CRC cases. In addition, they found that RAGE and adiponectin receptors (AdipoR1) could be involved in CRC, which concluded that AdipoR2 and GLO-I appeared as novel independent prognostic biomarkers for patients at early disease stage (76).

## Molecular Mechanisms

**Figure 2:**
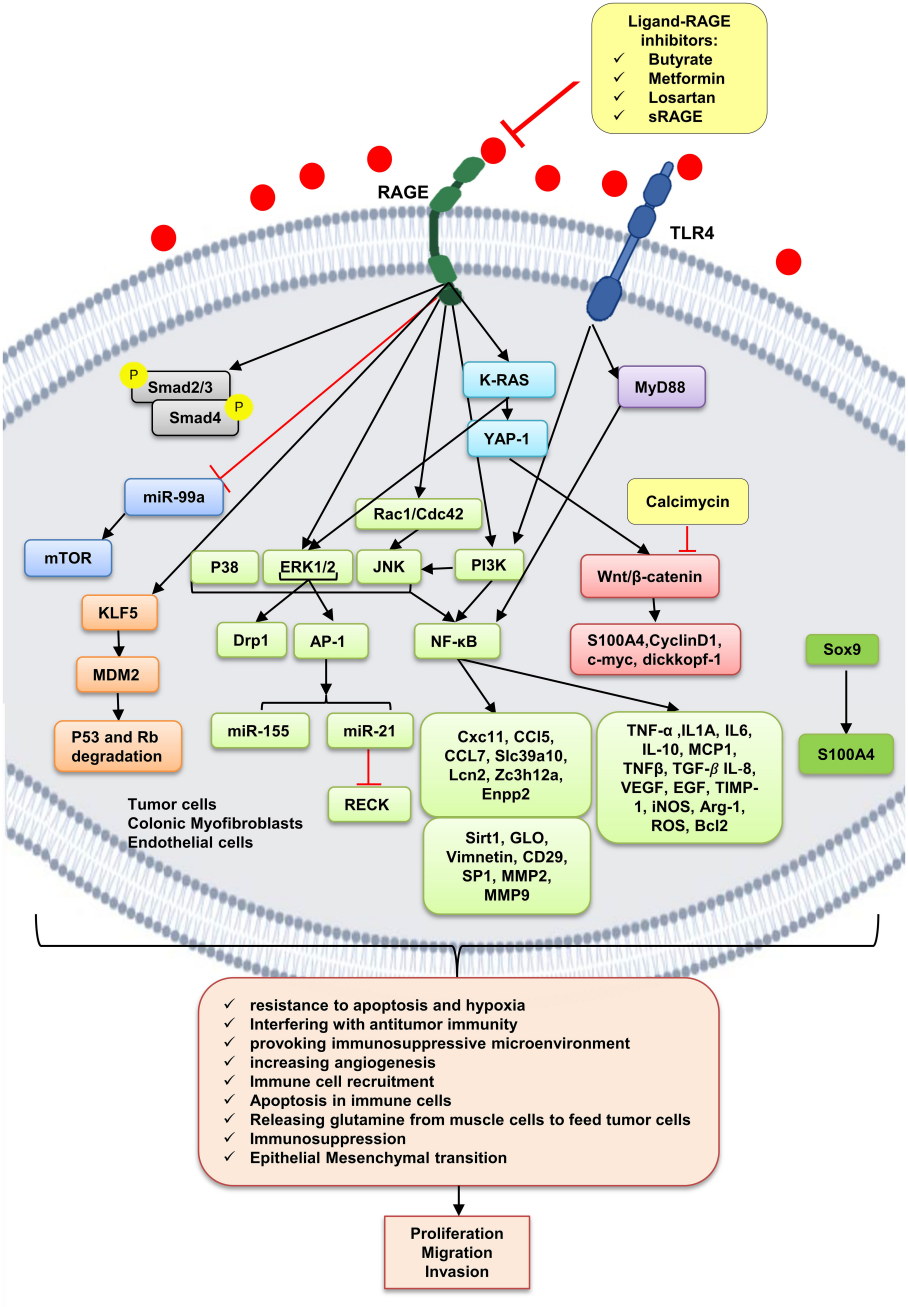
RAGE signaling pathway. The receptor of advanced glycation end-products (RAGE) is associated to triggering proinflammatory intracellular signaling cascades once it is engaged by RAGE ligands, leading to consistent and robust cellular responses. Once engaged, multiple signaling pathways are triggering, including K-RAS, erk1/2 MAP kinases, p38 and SAPK/JNK MAP kinases, Wnt/β-catenin pathway, NF-κB, AP-1, mTOR, Smad, PI3K, and the JAK/STAT pathways, with important downstream inflammatory and apoptotic marker secretions, which altogether leads to promotion of CRC. Butyrate, metformin, losartan, and sRAGE are RAGE–ligand interaction inhibitors.

As already mentioned, cancer cells, dying cancer cells after chemotherapy, express and release RAGE ligands, or AGE intake via diet can act in an autocrine and paracrine manner at tumor–host interface. According to Table 2, which includes studies on involvement of RAGE signaling pathways in colorectal cancer tumorigenesis, tumor growth, migration, and invasion, RAGE has been reported to drive assorted signaling pathways, including activation of activator protein 1 (AP-1), NF-κB, signal transducer and activator of transcription 3 (STAT3), SMAD family member 4 (Smad4), MAPKs, mammalian target of rapamycin (mTOR), phosphoinositide 3-kinases (PI3K), reticular-activating system (Ras), Wnt/β-catenin pathway, and glycogen synthase kinase 3β (GSK3β), and even microRNAs (Figure 2). However, most of these pathways were stimulated by different upstream kinase cascades or membranal adaptor signaling transducers. Here, mechanism by which RAGE–ligand interaction transduces its signaling is mentioned regarding ligands whose effects are studied in CRC such as HMGB1, S100A8/9, S100A4, S100P, AGEs, MG, AGE-BSA, and glucose-derived AGEs.

**Table 2:** Basic studies revealed the molecular mechanism involved in RAGE signaling pathway in CRC.

**References**	**Tissue/cell lines/animal model**	**Ligands**	**RAGE molecular mechanism**
Ang et al. (108)	SW837, SW480, Pan c-1/SWD20, SWK3	S100A8/A9	Smad4
Dahlamann et al. (40)		S100A4	MAPK/ERK and hypoxia
Deng et al. (72)	160 Tumors/SW1116, SW480, SW620, HCT116, Caco2, HT29, LoVo	AGEs-BSA	RAGE/ERK/SP1/MMP2
Fuentes et al. (8)	Caco-2 and SW480	S100P	ERK and NF-κB
Huang et al. (88)	30 Tumors/LoVo cells/male BALB/c nude mice (liver metastasis model) xenograft tumor model	HMGB1	NF-κB
Huang et al. (88)	SW480, SW620, LoVo, and LoVoOXR/tumor tissue	HMGB1	ERK1/2/Drp1
Huang et al. (121)	LoVo-induced MDSCs model/52 whole blood samples of CRC patients	S100A9	RAGE-p38 MAPK and TLR4–NF-κB
Ichikawa et al. (109)	Mice colitis-associated colon cancer/MC38 cells	S100A8/A9	MAPK and NF-κB
Kuniyasu et al. (156)	Rat peritoneal and human alveolar macrophages, WiDr, and PMA-U937 cells	HMGB1/amphoterin	Rac1 and JNK/SAPK
Kuniyasu et al. (2003)	Colo320, DLD1, WiDr, and TCO cells	(AGE-BSA)/amphoterin	ERK-1/2, Rac1 and AKT/MMP9, and NF-κBp65/NO
Liang et al. (48)	SW480, HCT116, SW620, LOVO, and Colo205 cell lines/45 CRC tissue samples	—	SP1/VEGF
Mercado-Pimentel et al. (161)	SW480 and LS174T	S100P	AP-1/miR-21/RECK
Onyeagucha et al. (162)	DLD-1, HEK-293T, LS174T, and SW480 cell lines	S100P	AP-1/miR-155
Qian et al. (163)	HCT116 and SW480 CRC cells	HMGB1	K-Ras/Yap1
Sack et al. (127)	HCT116 (HAB-68^mut^ and HAB-92^wt^)/xenograft mice	S100A4	Wnt/β-catenin
Sharma et al. (164)	HT-29, MCF-7, and A549	HMGB1	TLR4/MAPK and PI3K
Shen et al. (151)	CRC tissue samples/HCT 116/tumorigenesis mice model	S100P	SOX9/S100P
Shen et al. (152)	25 CRC tissue/LS174T and HCT116 cells/tumorigenesis mice model	S100P	MAPK/ERK
Turovskaya et al. (11)	9 Tumor tissue samples/RAGE^−/−^, RAGE^+/+^ C57BL/6 mice/CAC mice model (AOM+DSS)/HT-29, Caco-2 and CT-26	S100A8/A9	TLR4, glycans, and RAGE
Wang et al. (73)	HCT116/diabetic mouse model [ICR mice/streptozotocin (STZ)]	AGEs	KLF5/MDM2/p53 and Rb

### HMGB1

HMGB1–RAGE interaction at tumor cells acts through different molecular pathways, leading to tumor proliferation, migration, and invasion. It has been shown that RAGE knockdown mice developed decreased tumor number, size, and histopathologic grade. However, gain of function of RAGE and following HMGB1–RAGE interaction led to development of more intestinal adenomas and also increased the hepatic tumor loads. In this context, RAGE blocking by sRAGE or genetic deletion of RAGE (Rage^−/−^) reduced hepatic tumor rate, nodules, and load (87). In parallel, Apc^Min/+^/MyD88^−/−^ mice showed similar phenotype proposing that RAGE and MyD88 signaling share tumor-promoting mechanisms (45, 165).

**Figure 3:**
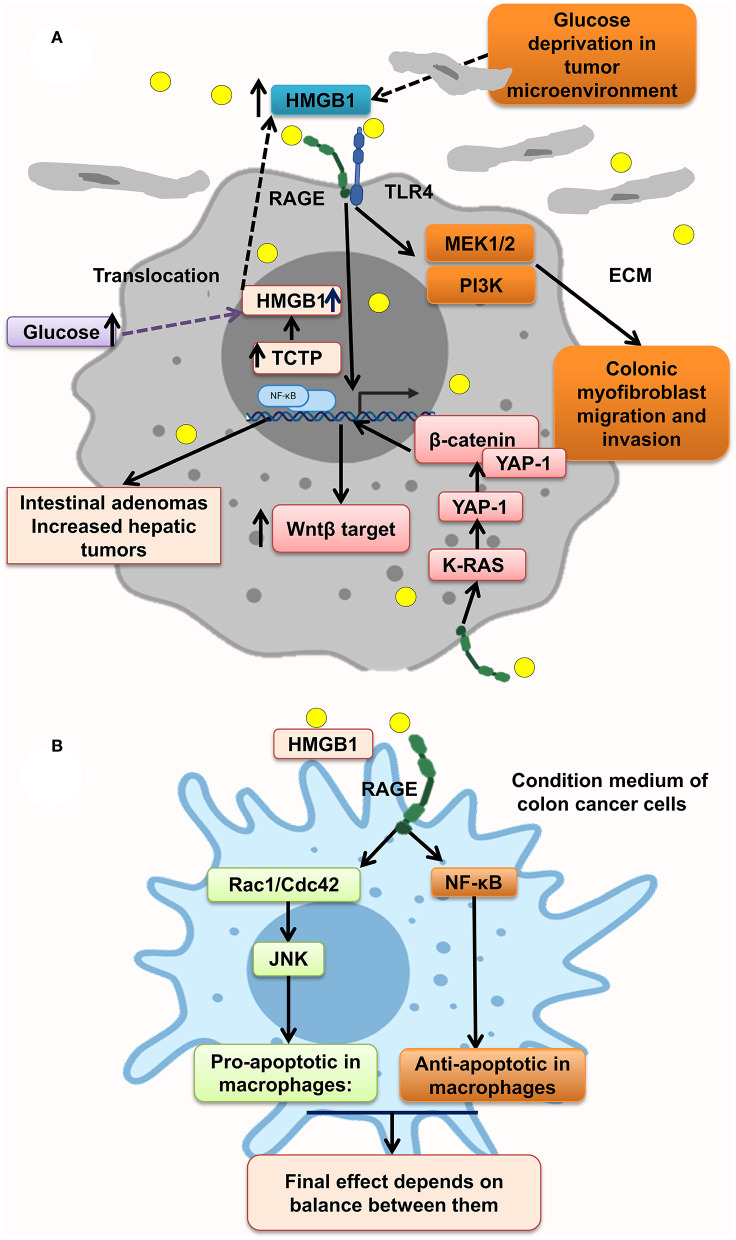
HMGB1 makes a metastatic microenvironment. **(A)** Glucose imbalance is an HMGB1 elevation trigger in tumor microenvironment by activating MAPK/ERK, Wnt/β-catenin, and PI3K pathways. **(B)** HMGB1–RAGE interaction results in both apoptosis and antiapoptosis via activation of, respectively, Rac1/Cdc42/JNK and NF-κB pathways.

Moreover, some protein overexpression has a positive effect on HMGB1 overexpression and translocation to cytoplasm and extracellular matrix of tumor cells, making an inflammation context microenvironment. Much evidence reveals that transnationally controlled tumor protein (TCTP), a very conserved and multifunctional protein, is involved in the tumorigenesis of several malignances (166). TCTP level in colon tumor tissues and HMGB1 level in serum of CRC patients were significantly increased. In line with this, TCTP overexpression on colon cancer cell lines resulted in the release of HMGB1 from the nucleus to the cytoplasm and into the extracellular space led to activation of NF-κB through the RAGE/TLR4/HMGB1 mediation, which simplified CRC cell invasion. This process is inverted by inhibition of the HMGB1-TLR4/RAGE–NF-κB pathway by specific antibodies significantly inhibiting the invasion (Figure 3A) (88). However, it has been shown that the NF-κB level in PMAU937 cells treated with HMGB1 was not considerably different from that in untreated cells. PMAU937 cells were high in phosphorylated levels of Rac1/Cdcd42 and JNK/SAPK result in proapoptotic consequence. Moreover, high NF-κB activation generates a survival signal to cells via antiapoptotic effect. The result depends on balancing of proapoptotic and antiapoptotic signals and other stimulating growth factors and cytokines. Also, they showed that HMGB1 is not a strong stimulant of nitric oxide (NO) in macrophages and so could not prompt cell death by NO (Figure 3B) (156). Glucose deprivation in tumor microenvironment is another stimulant of HMGB1 strong release of HMGB1 from several types of cancer cell lines under normal oxygen concentration, leading to activation of RAGE and TLR4, causing the activation of the MAPK/MEK1/2 and PI3K signaling pathways, leading to colonic myofibroblast migration and invasion (164). However, it has been indicated that, in the normal glucose state in NCM460 cells, proliferation is augmented by overexpression of HMGB1. Under a high glucose state, HMGB1 expression is elevated and released from cell nuclei into the cytoplasm and extracellular matrix of tumor (167) (Figure 3A).

Moreover, HMGB1–RAGE pathway stimulates Yap1 by direct association with K-Ras and results in CRC progression. Many pathways are activated through HMGB1–RAGE interaction including MAPK, Ras, Hippo, and Wnt/β-catenin signaling pathways as classic oncogenic pathways in CRC cells. Yap1 and β-catenin are physically linked to each other to enhance the Wnt target transcription in CRC cells, proposing that Wnt/β-catenin pathway possibly will contribute to CRC progression by RAGE activation trigger, which should be more elucidated in future studies (163) (Figure 3A).

**Figure 4:**
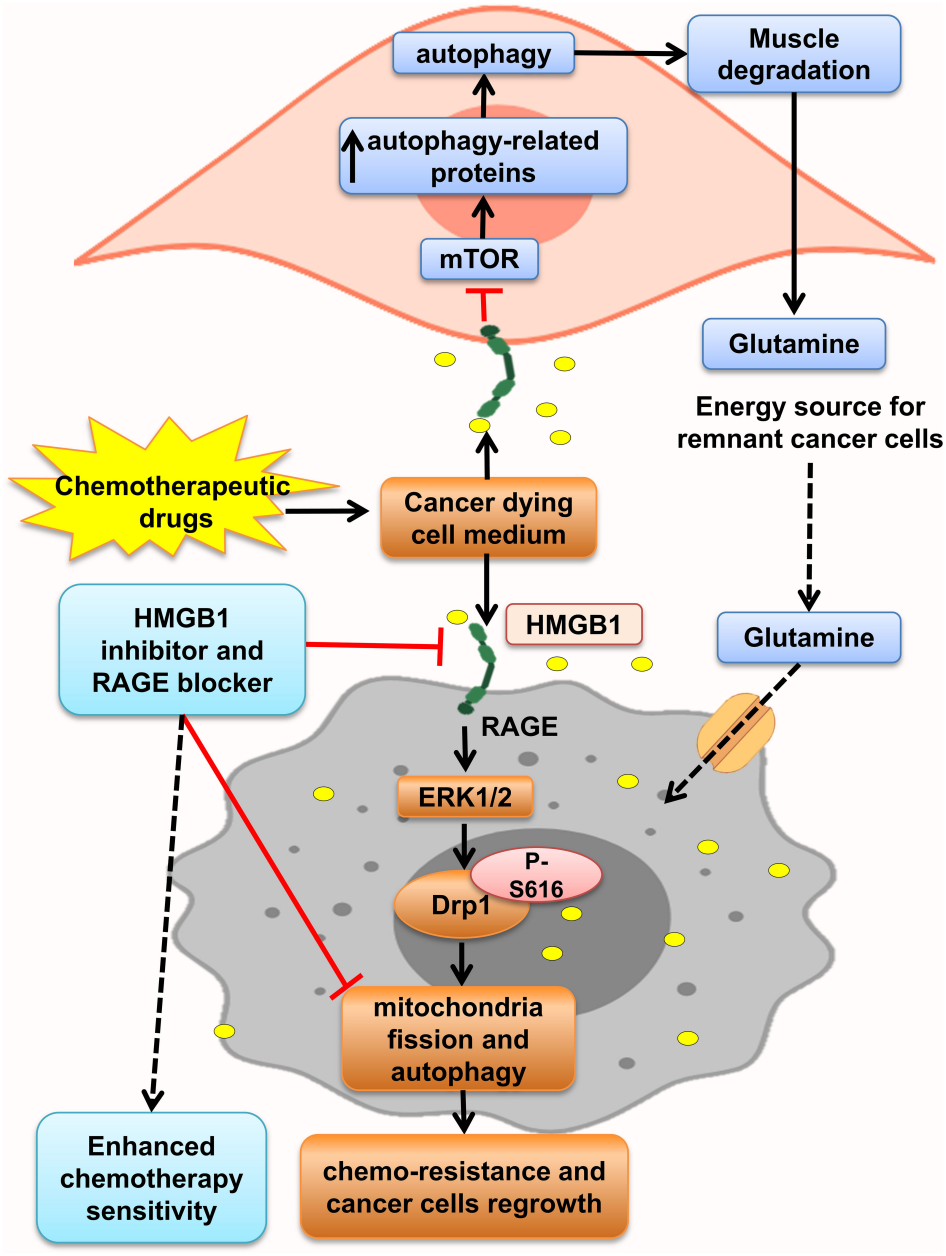
HMGB1 makes tumor cells resistant to chemotherapy. HMGB1 released from chemotherapy dying cancer cells in the tumor microenvironment and HMGB1–RAGE interaction on cancer cells lead to mitochondria fission, and autophagy via ERK1/2-dependent Drp1 phosphorylation enhances chemoresistance status. Besides, RAGE activation on muscle cells initiates muscle degradation, and releasing amino acids such as glutamine in the circulation and tumor environments provides energy source for sustaining growth of tumor cells.

Furthermore, HMGB1 and RAGE interaction activates MAPK signaling pathway through K-Ras, Rac1/JNK/SAPK, and ERK1/2/Drp1 phosphorylation and prompts CRC progression, chemoresistance and regrowth of cancer cells, and colonic myofibroblast proliferation, migration, and invasion (57, 156, 163, 164). Mitochondrial dysfunction has been shown to progress cancer cell proliferation, decrease apoptosis, and increase chemoresistance (168). HMGB1 is released from dying cells by chemotherapeutic drugs in conditioned medium and interacted with RAGE, signals ERK1/2 activation to phosphorylate Drp1 at residue S616, triggering mitochondria fission and autophagy, and promotes chemoresistance and regrowth in the surviving cancer cells, leading to poor survival outcome in locally advanced rectal cancer. HMGB1 inhibitor and RAGE blocker abolished Drp1 phosphorylation and considerably increased chemotherapeutic cure sensitivity by suppressing autophagy. Moreover, it has been shown that high phospho-Drp1Ser616 in CRC patients is related to a high possibility of developing tumor relapse. Moreover, RAGE-G82S polymorphism (rs2070600) has high ligand affinity and is related to high phospho-Drp1Ser616 in tumor microenvironment (57) (Figure 4).

Lue et al. indicated that after HMGB1 elevation in serum and colon of mice model of colon cancer, autophagy increased, which led to elevation of free amino acids in plasma, increased glutamine, and low PKM1 levels through activation of RAGE by HMGB1. HMGB1 enhanced autophagy in the muscle by decreasing active mTOR and augmentation of autophagy-related proteins and plasma glutamate. These aberrant effects were abolished by administration of an HMGB1-neutralizing antibody. So, this glutamine released from necrotic cells is used by the remnant cancer cell as an energy source and support their continuous growth (9). Moreover, Luo et al. in another study showed that necrosis inducer promotes cancer cell necrosis and releases HMGB1, which enhances regrowth and metastasis of remnant cancer cells by RAGE activation, but apoptosis inducers lack this effect. Thus, the outcomes proposed using apoptosis inducers more than necrotic inducers to prevent cancer relapse (169) (Figure 4).

Trophinin, a unique adhesion molecule, is expressed in human trophoblastic cells, with a pivotal role in trophoblast growth and invasion into the uterine wall to form the placenta (170). This process resembles tumor invasion into adjacent tissues. Trophinin is expressed in testicular germ cell tumors, leading to invasion and metastasis of tumor cells (171). Harada et al. indicated that SW480 cells that highly expressed trophinin and HMGB1 were more invasive than control. Small interfering RNA (siRNA) knockdown of trophinin reverses its effect in Colo320 cells. Positive correlation was found between HMGB1 protein expression in the nucleus and trophinin expression in tumor tissues, and its high expression in tumors of colon cancer patients is strongly linked to poor prognosis. Moreover, they investigated tumor tissues of 50 CRC patients and found HMGB1 and RAGE protein coexpression in 65.6% of trophinin-positive patients. Thus, it could be concluded that trophinin stimulates invasion via a mechanism involving HMGB1/RAGE (86).

Thus, as shown in Figures 3, 4, HMGB1–RAGE interactions on surface of tumor cells, inflammatory cells, and also muscle cells provide an inflammatory microenvironment that promotes chemoresistance and cancer cell regrowth via activating MAPK, PI3K, Wnt/β-catenin, and NF-κB signaling pathway. Also, blocking HMGB1–RAGE interaction could enhance chemotherapy sensitivity.

### S100A8/9

**Figure 5:**
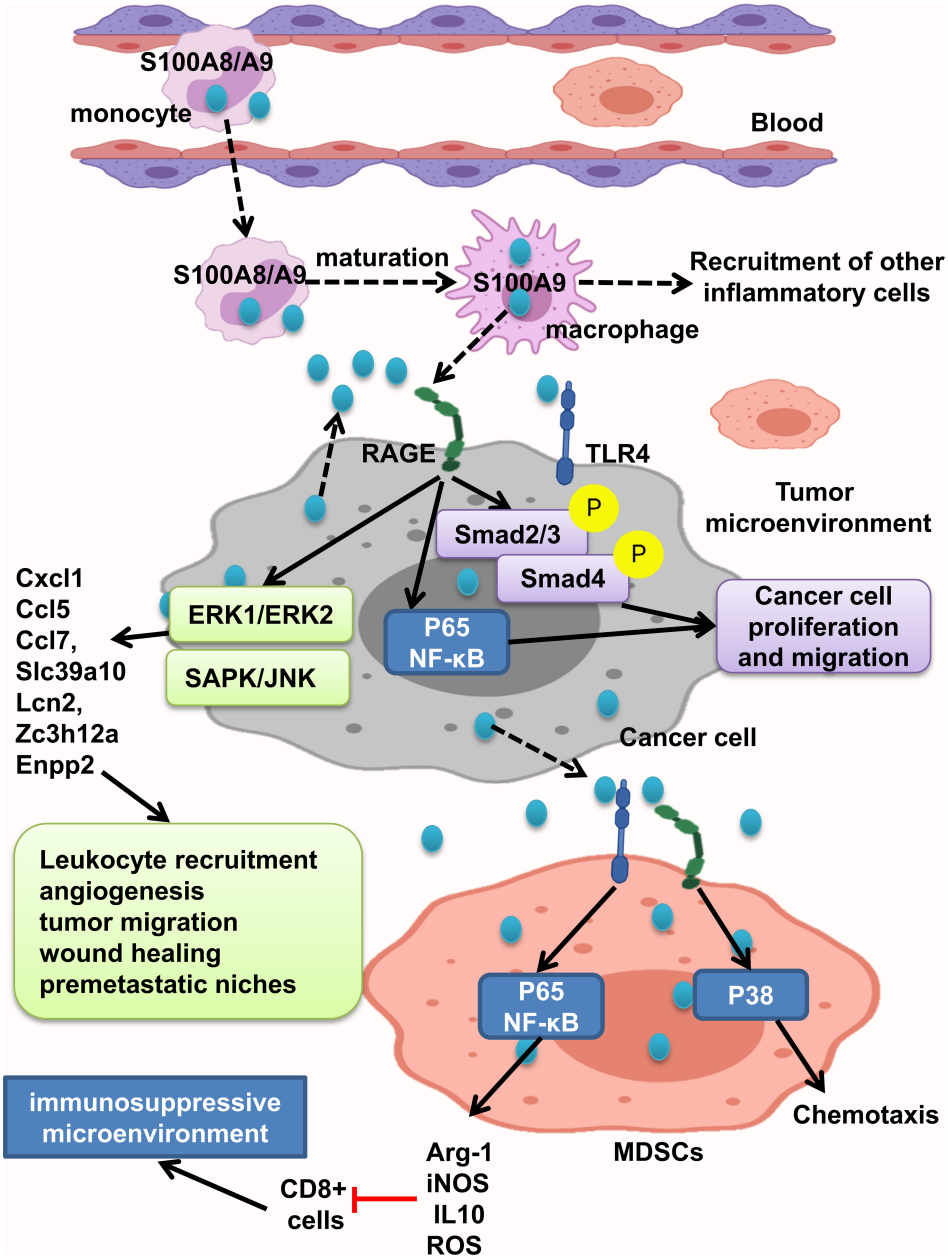
S100A8/A9 provides a premetastatic environment. S100A8/A9 expresses on tumor cells and also inflammatory cells. S100A8/A9–RAGE interaction results in activation of Smad, NF-κB, ERK1/ERK2, and SAPK/JNK pathways and increasing the leukocyte recruitment angiogenesis, tumor migration, and wound healing and consequently providing premetastatic niches. MDSCs will promote immunosuppressive microenvironment by activation of NF-κB pathway and also cause chemotaxis by P38 activation.

S100A8/A9 interaction with RAGE activates MAPK, Smad4, and NF-κB signaling pathways and develops intestinal adenoma, migration, invasion, and metastasis (11, 45, 108, 109, 121). Studies have shown that S100A8/A9 expression on myeloid cells is necessary for colon tumor growth (109). As recruited monocytes mature, they lose S100A8 and keep S100A9 expression and recruit more inflammatory cells, resulting in tumor cell proliferation and invasion. S100A8/9 boosted migration and proliferation in cells with or without Smad4 tumor cells. But, tumor cells with transient decrease of Smad4 do not react to S100A8, but not S100A9. Interaction of tumor cells with stromal myeloid cells and reaction to stromal chemokine are altered with loss of Smad4. S100A/S100A9 makes Smad4 signaling active as shown by phosphorylation of Smad2/3; RAGE blockage repressed this reaction (108) (Figure 5).

MDSCs suppress the antitumor immune response and enhance tumor growth and metastasis. It has been found that increased levels of S100A9 and MDSCs in tumor tissue and peripheral blood of CRC patients were related to neoplastic progression. Moreover, S100A9 regulates MDSC chemotaxis and activation, which induces immunosuppressive microenvironment. Elevated S100A9 in tumor microenvironment released from cancer cells, inflammatory cells, or MDSCs fuels RAGE- dependent p38 MAPK signaling cascade and also the TLR4-dependent NF-κB signaling cascade. Activation of p38 MAPK pathway led to MDSC chemotaxis, and NF-κB activation led to upregulation of immunosuppressive molecules inducible NO synthase (iNOS), Arg-1 and IL10 expression, and ROS production. Thus, assessing S100A9 and MDSCs in tumor tissue and also peripheral blood could help as a diagnostic marker of CRC progression (121). However, Ichikawa et al. indicated that S100A8/A9–RAGE and carboxylated glycan interaction activates phosphorylation of ERK1/ERK2 and SAPK/JNK MAPK in colon tumor cells, but not significant p38 phosphorylation, which led to elevation of Slc39a10, Cxcl1, Ccl5, Enpp2, Ccl7, Lcn2, Zc3h12a, and other genes, and stimulates recruitment of leukocytes, angiogenesis, migration of tumor cells, wound soothing, and creation of premetastatic microenvironment (109). Carboxylated glycans are expressed on a RAGE subpopulation on colon tumor cells and facilitate S100A8/A9 binding to RAGE results in NF-κB activation and cancer cell proliferation. S100A8/A9 could also activate TLR4 and increased tumorigenicity. Anticarboxylate glycan antibody blocks RAGE–ligand binding, downstream signaling, and tumor cell proliferation (11) (Figure 5).

In summary, tumor cells respond to S100A9 better than S100A8, and its interaction with RAGE triggers different signaling pathways in favor of inflammatory tumor microenvironment, including Smad, NF-κB, MAPK/ERK, and SAPK/JNK and P38, leading to leukocyte recruitment angiogenesis, tumor migration, wound healing, and premetastatic niches. Also MDSCs act as immune suppressor cells in tumor microenvironment and could be good tumor progression marker.

### S100A4

**Figure 6:**
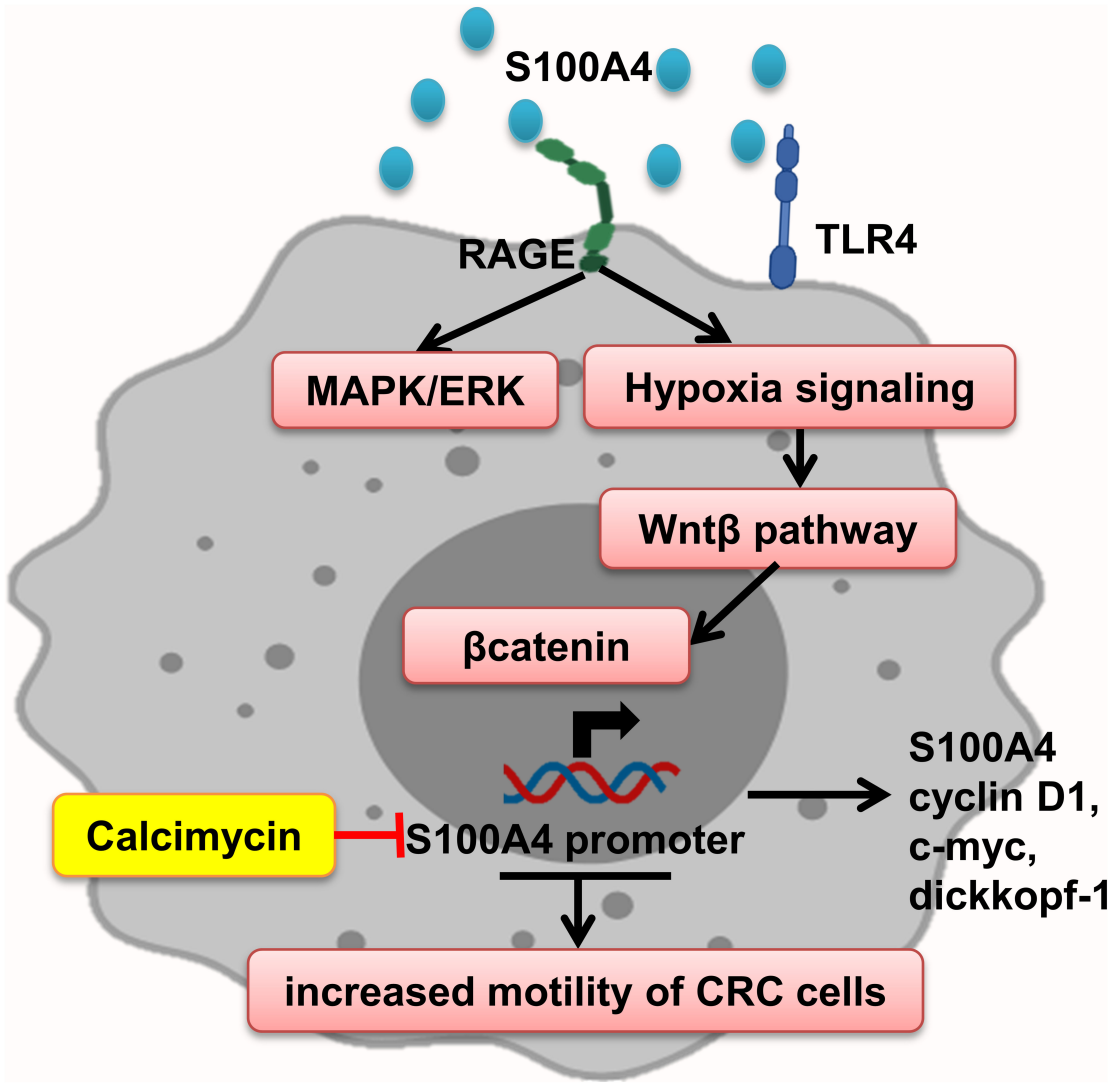
S100A4 increased motility of tumor cells. S100A4–RAGE interaction in tumor cells initiates activation of hypoxia, and MAPK/ERK signaling results in Wnt-β pathway activation and expression of β-catenin target genes, which promote elevated CRC cell motility. Calcimycin by suppressing the S100A4 promoter decreases S100A4 expression and other genes and inhibits Wnt-β pathway reverse S100A4 effect.

Interaction of S100A4–RAGE mediates MAPK/ERK and hypoxia signaling hyperactivation, leading to elevated CRC cell motility, which is significantly enhanced by adding rS100A4 (40). Sack et al. showed calcimycin inhibits promoter of S100A4 and decreases S100A4 expression and therefore impairs inducing of cell motility and metastasis, via suppression of Wnt/β-catenin pathway activity and expression of important β-catenin target genes such as S100A4, cyclin D1, c-myc, and dickkopf-1 (127) (Figure 6).

### S100P

**Figure 7:**
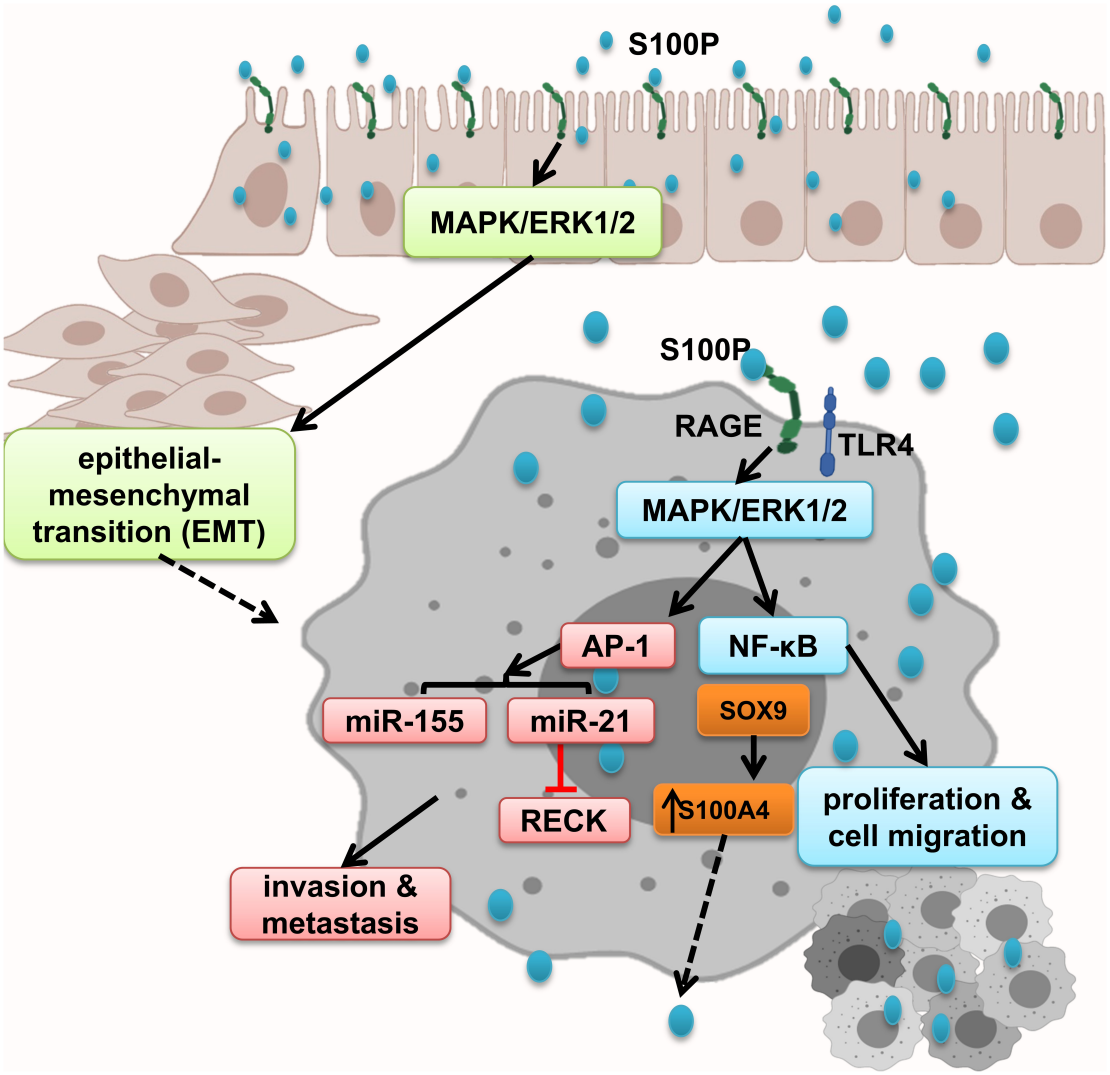
S100P promotes epithelial mesenchymal transition and tumor proliferation, migration, and invasion. S100P expresses only in malignant tissues and by activation of RAGE and downstream cascades including NF-κB and AP-1–dependent oncogenic mi-RNA activation, which altogether enhanced tumor migration and invasion.

While RAGE expresses in both normal and malignant colon specimens, S100P is expressed only in the malignant specimens and promoted cell growth; migration through RAGE activation, which leads to ERK1/2 phosphorylation; and NF-κB activation. In line with this, it has been shown that treatment of colorectal cancer cell lines with S100P increased proliferation and cell migration. Antagonism of RAGE blocked this interaction and irregular results (8). Moreover, Shen et al. showed that S100P and RAGE interaction leads to ERKs phosphorylation and promotes EMT (152). S100P expression is regulated by transcription factors and microRNA. Both SOX9 and S100P are overexpressed in colon cancer. SOX9, S100P transcription factor, increased S100P expression promote metastasis and invasion and result in low survival in colon cancer patients (151). Furthermore, studies show that microRNA dysregulation mediates cancer development and progression through activation of inflammatory cascades. MiR-21 is overexpressed in many types of human cancers, as well as colon cancer. S100P/RAGE interaction activates ERK1/2 phosphorylation, leading to the upregulation of oncogenic miR-21 via AP-1 signaling pathway, which leads to RECK suppression, and facilitates the onset of invasion and metastasis (161). Moreover, RAGE–S100P interaction activates MAPK pathway, and AP-1 leads to expression of oncogenic miR-155 expression, implicated in several malignancies such as colon cancer by linking inflammation and cancer. Prominent levels of miR-155 have been detected in primary colon cancers and metastatic lesions. It has been shown that exogenous S100P cannot activate MAPK-dependent miR-155 after suppressing activation of AP-1 by inhibiting MEK activation or genetically inhibiting c-Jun activation. Thus, miR-155 was regulated by MAPK kinase and also, to a lesser extent, NF-κB (162, 172–175) (Figure 7).

In conclusion, S100P is only expressed in malignant tissues that result in EMT by activating MAPK/ERK1/2 and S100P accumulation that initiates NF-κB activation and AP-1–dependent oncogenic mi-RNA activation, which altogether enhanced tumor migration and invasion.

## Therapeutic Strategies Against RAGE in CRC

As shown in many studies mentioned in this review investigating the role of RAGE by its blockage through siRNA and antibodies and antagonists treatment, RAGE activation stopped, as well as underlying mechanisms leading to tumorigenicity. Also, it has been indicated that antibodies against RAGE ligands can decrease the risk of CRC (176–180). This, as mentioned before, the effect of prolonged RAGE blockade in human subjects is important because RAGE plays vital roles in normal physiology. Moreover, 30 percent of IBD patients did not respond to anti-TNF-α and other therapeutic options such as antibodies against cytokines that promote infection in patients (181, 182). In this regard, few studies investigated the effect of less toxic therapeutic agents such as ligand-scavenging components such as phytochemicals. Flavonoids, stilbenes, catechins, phenolic acid, anthocyanin, genistein, and curcumin are mentioned as AGE formation inhibitors and lead to decreased risk of diabetes and CRC (183–186). Fluoroquinolones are promising therapeutic agents for increasing the related α-dicarbonyl (AGE precursors) scavengers as to protect cells against carbonyl stress (187). Heat-stabilized rice bran consumption in colorectal patients has been indicated in the modulation of stool metabolites such as AGEs (188). Also, medicinal plants such as *Carpobrotus edulis* (African) as well as *Castanea mollissima* Blume (Chinese) work against protein glycation and colon cancer by inhibition of AGE production (189, 190). Therefore, novel phytochemical components by inhibiting RAGE activation could be less toxic and safer to approach against cancer.

## AGEs-RAGE Interaction Regard to T2D and CRC Association

Lifestyle in industrialized countries, such as physical inactivity and diet, leads to increased mortality and threat to public health in the world. Many studies have revealed that obesity, chronic inflammation, aging, and insulin resistance are associated risk factors inducing CRC development (191–193). Moreover, T2D could decrease the survival of CRC patients. The evidence has shown that elevated insulin concentration is able to develop CRC cell growth, but the mechanism and molecular link between CRC and T2D are controversial (194–196). However, metabolic syndrome, especially insulin resistance and hyperglycemia, could explain the relationship between these diseases (197, 198). Nevertheless, there was a missing link in related signaling pathways. Yamagishi et al. hypothesized that RAGE and enhanced serum AGEs were the bridge between associated diseases and increase the risk of colorectal cancer in patients with diabetes (199). Upregulated RAGE and its ligands have been indicated in different inflammatory conditions, including IBD, diabetes, and cancer (54). Based on these studies, RAGE has essential roles in tumorigenesis, and metastasis–ligand–RAGE axis plays a vital role in sophisticated paracrine and autocrine manners within the tumor microenvironment to promote cell migration, invasion, and survival (27, 200, 201). Particularly, Liu et al. in 2014 enrolled 21 CRC patients with metabolic syndrome, and they found that IL-6, IGF-1, and VEGF-1 circulation was unchanged. However, IGF-1R and RAGE expressions were increased in tumor tissues; also, fasting insulin levels in CRC patients with metabolic syndrome were significantly enhanced (149). Moreover, Deng and colleagues have shown the risk of CRC in diabetic patients significantly increased, and glucose-derived AGEs were increased in CRC patients serum and activate RAGE/ERK/SP1/matrix metallopeptidase-2 (MMP2) cascade in cancerous tissues promote the invasion and metastasis of CRC. This is reversed by RAGE blocking Ab and SP1-specific siRNA. Deng et al. demonstrated the expression of specificity protein 1 (Sp1), MMP2, and RAGE predominately increased in cancerous tissues (72). AGE and RAGE were unregulated in the colonic mucosa of azoxymethane (AOM)-injected F344 rats, especially in rats fed with high-LA and high-glucose diets, and this upregulation induced continuous ROS production and was associated with increased ACFs and carcinomas in the rat colon. Moreover, treatment with metformin and losartan inhibited AGE and reduced cancer multiplicity in the rats that received LA or normal diet and glucose drink in comparison to the control rats. Losartan showed mild inhibition of AGE formation in a non-cellular system, suggesting that losartan possesses a weak antioxidative effect independent of its antiangiotensin activity. RAGE-associated genes were upregulated including MMP9, VEGF, iNOS, and BCL2 in the carcinomas. The phosphorylation of ERK1/2, Rac1, and AKT and the production of MMP9 are increased more by HMGB1 than by AGE, whereas the production of iNOS and NF-κB is increased more by AGE than by HMGB1. AGE–RAGE induced by high-LA and high-glucose diets substantially enhances colon cancer development (41, 202, 203). Moreover, some transcription factors, such as SP1, p53, NF-κB, and KLF7, could regulate oncogenic gene. MDM2 and MDM2 overexpression is a candidate biological link between T2D and colon cancer development. Wang et al. in 2018 indicated that diabetes increases the level of AGE, which enhances the expression of MDM2 via transcription factor KLF5 in colon cells. MDM2 binds directly to cancer suppressors; p53 and Rb promote their inactivation and degradation (73). Also, Kuniyasu et al. claimed that invasion and metastasis are associated with raised expression of RAGE and amphoterin in 119 non-diabetic patients with CRC (156). Besides, several studies have suggested inflammation has a prominent role in IBD that is associated to amplified risk of CRC (204–206). Nevertheless, Kong et al. showed that in a total of 1,055 CRC cases glycer-AGEs levels were raised; however, this was not related to colon risk. As a result, AGEs were not associated with amplified risk of colorectal cancer (69). AGEs are able to affect the antioxidant defenses and augmented ROS and apoptosis (207, 208). However, Kucukhuseyin et al. detected increased level of advanced oxidation protein products and protein carbonyl in CRC patients, and the levels of antioxidants such as Cu-Zn SOD and total thiol were lessened (148). In the other study, Sakellariou et al. in 2016 claimed that there was a relationship between RAGE and detoxification enzyme GLO-I and adiponectin receptors (AdipoR1, AdipoR2) in CRC. They investigated 133 primary CRC cases and found that RAGE and AdipoR1 might be involved in CRC; also, the upregulation of AdipoR2 and GLO-I reduced survival in the whole cohort and early-stage cases. Notably, redox levels as biomarkers in CRC predominately were changed. These data were in agreement with those of Thornalley et al. (76, 209). In general, the accumulation AGEs and binding to RAGE lead to activation and oligomerization of various inflammatory and oxidative stress.

The mechanism of abnormal epithelial proliferation under the diabetic state is unclear. Several literatures have shown that gut microbiota has a pivotal role in intestinal disease (210, 211). Moreover, the level of HMGB1 is elevated in the serum of these related diseases. Interestingly, Wang et al. in 2019 have attempted to observe the association of hyperproliferation of colonic epithelium under a diabetic state with RAGE and HMGB1; besides, they investigated the influence of butyrate on the proliferation of NCM460 cells. Notably, Wang and colleagues indicated that high glucose state increased proliferation by overexpression of HMGB1 and RAGE. On the other hand, butyrate could be suppressed, enhancing proliferation by targeting HMGB1. These data have shown that butyrate inhibited the expression of RAGE and prevented AGEs accumulation (167). Arabiyat et al. synthesized antioxidative and anti-inflammatory molecules, including fluoroquinolones and triazolofluoroquinolones, which could be suppressed in the AGEs binding to RAGE, and accumulation of AGEs was inhibited as a consequence. Moreover, this study evaluated the potential antiproliferative efficacy against colorectal cancer cell lines, as well as obesity and diabetes (187). Furthermore, Zhang et al. have explored *C. mollissima* Blume (Chinese chestnut) enriched by flavonoids, and polyphenolic acids might have antidiabetic complications and anticancer activities (190). A recent study has shown that some mi-RNA levels are involved in CRC and T2D, and lifestyle could influence these diseases. A study by Zhu et al. in 2019 revealed the relationship between miR-99a and mTOR in 20 patients with CRC and T2D. They found AGEs could impair the miR-99a, leading to overactivated mTOR signaling. In addition, microarray analysis indicated this mi-RNA might be a biomarker and therapeutic target (175).

Recently, elevated levels of MG were found in mammals consuming a Western-style diet. MG is derived from glycolysis, lipid peroxidation, and protein degradation. Many studies demonstrated that MG-induced carbonyl stress can lead to oxidative stress and inflammation, and it was highlighted in the pathogenesis of obesity, diabetes, and metabolic syndrome (75). Exposure to MG led to increases in the serum low-density lipoprotein/high-density lipoprotein ratio, fecal bile acid, and foci of aberrant crypts in the colon (ACFs) and aberrant crypts (ACs) in the colon of AOM-treated mice, and oxidative and carbonyl stress (MG, AGEs, and malondialdehyde levels) inflammatory mediators (IL-6 and RAGE) in tumors of CT26 isografts in mice. Also, increased activation of signaling pathways that modulate cell survival and proliferation (i.e., the ERK/p38 MAPK, and PI3K/GSK3β/mTOR pathways) and increased level of GLO and Sirt1 were observed in MG-induced cells with increased proliferative and migratory activities as well as stem-like properties (increased CD29). Excitingly, boosted expression or activation of proteins that modulate cell survival, proliferation, or migration/invasion (such as vimentin) was also observed. MG-induced carbonyl stress may be the pivotal agent involved in colon cancer progression. Furthermore, high level of MG adducts and low GLO-I activity in high stage tumors promote CRC development and tumor growth, which is reversed by carnosine, a potent MG scavenger (74, 75).

Thus, it could be concluded that different AGE subtypes and derivatives are accumulated in T2D and by interaction with RAGE trigger detrimental procedures, leading to increased inflammation and ROS production end in microenvironments, which increases the risk of CRC.

## Concluding Remarks

At present, convincing evidence indicates RAGE activation makes a tumor-promoting milieu, which in turn favors proliferation and survival of colorectal cancer cells. In this circumstance, developing investigational data propose that the multiligand–RAGE axis may be a significant contributor to inflammation-related tumorigenesis through different signaling mechanisms including activation of AP-1, NF-κB, STAT3, SMAD family members, MAPKs, mTOR, PI3K, Ras, Wnt/β-catenin, and GSK3β and even microRNAs, which epigenetically regulates the expression of RAGE ligands. These contain the activation of crucial processes that might help in the resistance to apoptosis and hypoxia, getting involved in antitumor immunity, provoking immunosuppressive microenvironment, increasing angiogenesis, and helping invasiveness. So, multiligand–RAGE axis has developed a very interesting target for pharmacological interventions directed to block RAGE–ligand interactions at the receptor level and also inhibited by scavenging its ligands. But as the function of RAGE and its ligands is tissue specific and has a role in normal physiology, as well as different pathologies, approaching appropriate therapeutic strategies are important. However, various questions remain unanswered to completely comprehend the role of this receptor in tumor–host cells interactions, particularly the other ligands that activate RAGE, the recruitment and activation of MDSCs, and the cross-talk between RAGE and TLRs.

## Author Contributions

FA-F: conception, providing the data and design, and manuscript writing. KG, MN-E, AG, and MH: conception and final approval of manuscript. NA: conception, providing the data and design, and manuscript writing. All authors contributed to the article and approved the submitted version.

## Conflict of Interest

The authors declare that the research was conducted in the absence of any commercial or financial relationships that could be construed as a potential conflict of interest.

## Abbreviations

AdipoR: Adiponectin Receptors
AGEs: Advanced Glycation End-Products
AD: Alzheimer's Disease
(AP-1): Activating Protein-1
CD28: Cluster of Differentiation 28
CRC: Colorectal Cancer
CTLA4: Cytotoxic T-lymphocyte-Associated Protein 4
Cu–Zn: Copper, Zinc
CVDs: Cardiovascular Diseases
SOD: Superoxide Dismutase
Drp1: Dynamin-Related Protein 1
EGF: Epidermal Growth Factor
EMT: Epithelial–Mesenchymal Transition
EN-RAGE: Extracellular Newly Receptor for Advanced Glycation End-Products
FN3K: Fructosamine 3 Kinase
GSK3β: Glycogen Synthase Kinase 3β
HMGB1: High-Mobility Group Box 1
IBD: Inflammatory Bowel Disease
Ig: Immunoglobulin
IGF-1: Insulin-Like Growth Factor-1 (IGF-1)
IL-8: Interleukin-8
LHP: Lipid Hydroperoxide
LARC: Locally Advanced Rectal Cancer
MAPKs: A Mitogen-Activated Protein Kinase
MCP-1: Monocyte Chemoattractant Protein-1
mTOR: Mammalian Target of Rapamycin
MyD88: Myeloid Differentiation Primary Response 88
NF-κB: Transcription Factor Nuclear Factor-κB
NO: Nitric Oxide
PD: Parkinson's Disease
PI3K: Phosphoinositide 3-Kinases
RAGE: Advanced Glycation End Products Receptor
Ras: Reticular Activating System
ROS: Reactive Oxygen Species
RE: Radioembolization Therapy
siRNA: Small Interfering RNA
Smad4: SMAD Family Member 4
SNPs: Single-Nucleotide Polymorphisms
SP-D: Pulmonary Surfactant-Associated Protein D
STAT3: Signal Transducer and Activator of Transcription 3
TCTP: Translationally Controlled Tumor Protein
T2D: Type 2 Diabetes
TIMP-1: Tissue Inhibitor of Metalloproteinases 1
TLR4: Toll-Like Receptor 4
TNF-α: Tumor Necrosis Factor-α
TNFR2: Tumor Necrosis Factor Receptor 2
T-SH: Total Thiol
VEGF: Vascular Endothelial Growth Factor.
